# IRF-5 Expression in Myeloid Cells Is Required for Splenomegaly in *L. donovani* Infected Mice

**DOI:** 10.3389/fimmu.2019.03071

**Published:** 2020-01-21

**Authors:** Linh Thuy Mai, Mélina Smans, Sasha Silva-Barrios, Aymeric Fabié, Simona Stäger

**Affiliations:** Centre Armand-Frappier Santé Biotechnologie, Institut National de la Recherche Scientifique (INRS), Laval, QC, Canada

**Keywords:** *Leishmania*, IRF5, myeloid cells, splenomegaly, Th1, LysM-cre

## Abstract

Persistent *Leishmania donovani* infection is characterized by chronic inflammation, immune suppression, and splenomegaly. We have previously reported that the transcription factor interferon regulatory factor 5 (IRF-5) is largely responsible for inducing the inflammatory response and maintaining protective Th1 cells following *L. donovani* inoculation in mice. However, the cellular source responsible for these effects is yet unknown. In this study, we investigated the role of IRF-5 in myeloid cells during experimental visceral leishmaniasis (VL). First, we show that the *LysM-Cre* mouse model is not suited for investigating gene expression in splenic myeloid cells during experimental VL. Using the *Cd11c-Cre* mouse model, we demonstrate that *Irf5* expression in CD11c^+^ cells (monocytes, dendritic cells, activated macrophages) is essential for inducing splenomegaly and for recruiting myeloid cells to the spleen, but it is not required for the development or maintenance of parasite-specific IFNγ-producing CD4 T cells. CD11c-specific *Irf5*^−/−^ mice are more resistant to *L. donovani* infection, suggesting that the induction of splenomegaly is detrimental to the host.

## Introduction

The Leishmaniasis are a set of vector-borne parasitic diseases that affect an estimated 12 million people worldwide. Disease clinical manifestations range from self-healing cutaneous lesions to life-threatening visceral infection. The visceral form of the disease has a fatality rate of 75–95% if left untreated (1) and is characterized by weight loss, irregular bouts of fever, anemia, hepatosplenomegaly, hypergamaglobulinemia, and immunosuppression. Experimental infection of mice with *Leishmania donovani*, a causative agent of visceral leishmaniasis (VL), also results in hepatosplenomegaly, but it is not fatal (2). In mice, parasite growth in the liver is controlled by protective CD4 and CD8 T cell responses producing IFNγ (3). In contrast, infection persists in the spleen and the bone marrow.

The chronic stage of experimental VL in the spleen is characterized by a chronic inflammatory environment, with all the consequences that this entails: tissue disruption, hypoxia, and immunoregulatory responses (2, 4, 5). *Leishmania* parasites survive very well in this environment, whereas protective T cell responses are inhibited in various ways. For instance, at d28p.i., CD8 T cells are dysfunctional and exhausted (6, 7) and CD4 T cells fail to expand (8), die by TRAIL-mediated apoptosis (9), and are suppressed by IL-10 (10–13) and by myeloid-derived suppressor cells (14).

Splenomegaly and chronic inflammation are associated with parasite persistence during chronic VL. Several cell populations contribute to splenomegaly, but myeloid cells, in particular, are progressively recruited to the spleen over the course of infection (14). Indeed, *L. donovani* induces the heightened release from the bone marrow of inflammatory monocytes (15). These cells display a regulatory phenotype and are more permissive to infection, favoring parasite growth and persistence (14–16). The inflammatory response during VL appears to require the activation of the transcription factor Interferon Regulatory Factor 5 (IRF-5). IRF-5 function has been mainly described in antigen-presenting cells, where it promotes the transcriptional activation of genes encoding for IFN-I and pro-inflammatory cytokines, such as TNF, IL-12, and IL-6 (17, 18). In human, IRF-5 polymorphisms are associated with various autoimmune inflammatory disorders (19–22). In mice infected with *L. donovani*, IRF-5 governs the inflammatory response (23), but it is also responsible for CD4 T cell death during chronic infection (9). Indeed, *Irf5*^−/−^ mice fail to develop splenomegaly and to develop protective Th1 responses following *L. donovani* infection (23). Nevertheless, the cellular source required for promoting IRF-5-dependent inflammation and sustaining Th1 responses during experimental VL is yet unknown.

In this study, we investigated the role of IRF-5 in myeloid cells following *L. donovani* infection in mice. We show that *LysM-Cre* mice are not a good model for investigating gene expression in splenic myeloid cells during experimental VL. We also demonstrate that *Irf5* expression in CD11c^+^ cells is essential for inducing splenomegaly, but it is not required for the development or maintenance of parasite-specific IFNγ-producing CD4 T cells.

## Materials and Methods

### Mice and Parasites

B6.129S7-*Rag1*^*tm*1*Mom*^ and cre recombinase-expressing mice were purchased from The Jackson Laboratory. Mice with a targeted *Irf5* mutation in myeloid and in CD11c^+^ cells were generated by crossing *Irf5*^flox/flox^ mice with mice expressing the cre-recombinase under the LysM and the CD11c promoter, respectively. All mice were housed at the INRS animal facility under specific pathogen-free conditions and used at 6–10 weeks of age. *Leishmania donovani* (strain LV9) were maintained by serial passage in B6.129S7-*Rag1*^*tm*1*Mom*^ mice; amastigotes were isolated from the spleen of infected animals (24). Mice were infected by injecting 2 x 10^7^ amastigotes intravenously via the lateral tail vein. Splenic parasite burden were determined by examining methanol-fixed, Giemsa stained tissue impression smears. Data are presented as Leishmania Donovani Units (LDU) (25).

### Ethic Statement

Experiments involving mice were carried out under protocols approved by the Comité Institutionnel de Protection des Animaux of the INRS-Institut Armand Frappier (1510-02, 1602-02). These protocols respect procedure on good animal practice provided by the Canadian Council on animal care.

### Flow Cytometry

Mice were euthanized at indicated time points. Mononuclear cells were purified from the liver and CD4 T cell responses were analyzed as previously described (14). Briefly, hepatic mononuclear cells were restimulated with bone marrow-derived dendritic cells, pulsed with fixed parasites, and directly incubated at 37°C in the presence of 1/1000 Brefeldin A (GolgiPlug™, BD Biosciences). Cells were then stained with anti-CD4-FITC (BD Pharmingen^TM^, clone GK15), anti-CD3-BV421 (BD Biosciences, clone 14S-2C11), followed by anti-IFN-γ-APC (BD Pharmingen^TM^, clone XMG1.2) after permeabilization with 0.1% saponin. Myeloid cells were stained with anti-CD11b-Pacific Blue (BD Horizon^TM^, clone MI/70), anti-MHC-II-FITC (BD PharmingenTM, clone 2G9), anti-Ly6C-PerCP (Biolegend, clone HK1.4), anti-Ly6G-PE (Biolegend, clone 1A8), anti-F4/80-PECy7 (Biolegend, clone BM8), and anti-CD11c-APC (eBioscience, clone N418). Flow cytometric analysis was performed with a BD LSRFortessa cell analyzer (Becton Dickinson). Samples were analyzed with Flowjo software.

### Enrichment of Splenic Myeloid Cells

CD11b^+^ cells were purified using magnetic cell sorting (MACS) from spleens of infected and naïve mice previously digested with collagenase D, following manufacturer's instructions (Miltenyi Biotec). The purity of the samples comprise between 90 and 93%.

### Real-Time PCR Analysis

Real-time PCR (Stratagene mx3005p Real time PCR System) was used to analyze transcripts levels of HPRT, HIF-1α, and IRF-5. Total RNA was insolated using RNeasy (Qiagen) to perform real-time RT-PCR. cDNA was prepared using 500 ng of total RNA using High Capacity cDNA Reverse Transcription Kit (Bio Rad). Real time PCR was performed using standard cycle of amplification. All PCRs were carried out with the Stratagene mx3000p real-time PCR system. *Irf5, Hprt*, and *Hif1a* were amplified using primers as previously described (9, 14). Data were normalized to HPRT and expressed as fold increase to naive controls.

### Statistical Analysis

Data were analyzed using Graphpad Prism (GraphPad Software). Statistical significance was assessed using two-way ANOVA. Differences were considered to be statistically significant when *p* < 0.05. All experiments were conducted independently at least three times.

## Results

### *Irf5*-Deletion in Myeloid Cells Does Not Affect the Hepatic and Splenic Parasite Burden

**Figure 1:**
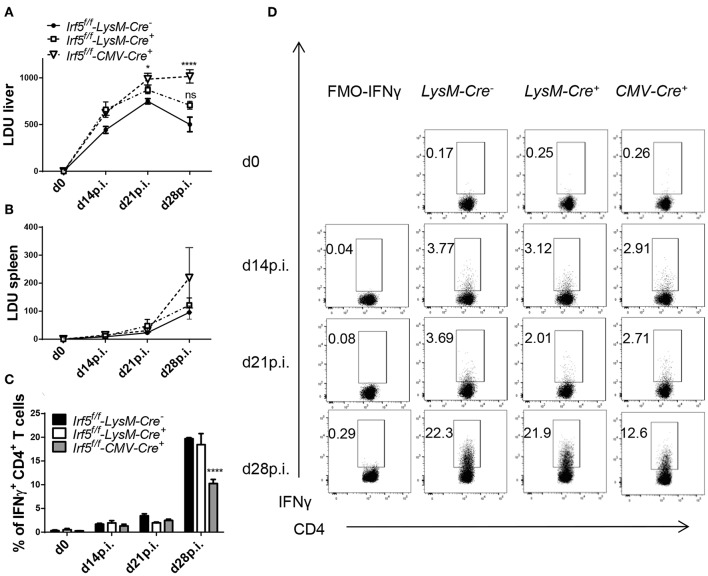
*Irf5*^*f*/*f*^-*LysM -Cre*^+^ mice are not more susceptible to *L. donovani* infection than *Irf5*^*f*/*f*^ -*LysM-Cre*^−^ mice. *Irf5*^*f*/*f*^ **-***LysM-Cre*^−^, *Irf5*^*f*/*f*^ **-***LysM-Cre*^+^, and *Irf5*^*f*/*f*^ **-***CMV-Cre*^+^ mice were infected with *L. donovani* and euthanized at d14, 21, and 28 p.i. Leishmania Donovan Units (LDU) were calculated from hepatic **(A)** and splenic **(B)** impression smears for the three group of mice. Graphs show representative scatter plots **(C)** and percentages **(D)** of CD4 T cells producing IFN-γ following *L. donovani* infection in the liver from three groups of mice. Error bars indicate mean ± SEM, *N* = 4, one of 5 independent experiments is shown; ns, not significant, *p < 0.05, ****p < 0.0001.

We have previously reported that the transcription factor IRF-5 majorly contributes to the inflammatory response following *Leishmania donovani* infection in mice (23). Indeed, *L. donovani*-infected *Irf5*^−/−^ mice not only failed to develop splenomegaly, but also displayed severely impaired parasite-specific Th1 responses. This resulted in higher hepatic parasite burden, but no differences were observed in the spleen (23). In the present study, we sought to identify the contribution of myeloid cell-derived IRF-5 expression to the immune response against *L. donovani*. To this end, we generated myeloid cell-specific *Irf5*^−/−^ mice by crossing *Irf5*^*flox*/*flox*^ with *LysM-Cre* mice and infected *Cre*^+^ (myeloid cells are IRF-5 deficient) and *Cre*^−^ (myeloid cells are IRF-5 sufficient) littermates with *L. donovani*. Because *Irf5*^−/−^ were shown to have a spontaneous mutation in *Dock2* that affected pDC and B cell development (26), we generated new *Irf5*^−/−^ mice by crossing *Irf5*^*flox*/*flox*^ with *CMV-Cre* mice. These mice were used as total *Irf5*^−/−^ controls in all experiments. First, we assessed the hepatic and splenic parasite burden. As expected, *L. donovani* infection in the liver was significantly exacerbated in *Irf5*^*flox*/*flox*^- *CMV-Cre*^+^ mice compared to infected *Irf5*^*flox*/*flox*^- *LysM-Cre*^−^ control mice (Figure 1A). In contrast, no differences were observed between the *Irf5*^*flox*/*flox*^- *LysM-Cre*^+^ and *Cre*^−^ groups. As previously reported (23), the absence of IRF-5 expression in all cells did not affect the splenic parasite load (*Irf5*^*flox*/*flox*^- *CMV-Cre*^+^); moreover, *Cre*^+^ mice had similar parasite numbers in the spleen than their *Cre*^−^ counterpart (Figure 1B).

### Myeloid-Specific *Irf5^−/−^* Mice Develop Similar Th1 Responses Than IRF-5-Sufficient Mice

A hallmark of *Irf5*^−/−^ mice is their incapacity to generate strong Th1 responses following *L. donovani* infection (23). Krausgruber et al. have also reported that IRF-5 is required to promote inflammatory macrophages and induce Th1 responses (27). Hence, we evaluated parasite-specific IFNγ^+^ CD4 cell responses at various time points of *L. donovani* infection in total *Irf5*^−/−^ (CMV-*Cre*^+^), in myeloid cell-specific *Irf5*^−/−^ (*Cre*^+^), and in IRF-5 sufficient (*Cre*^−^) mice. As shown in Figure 1C and in agreement with the literature (23), in *Cre*^−^ control mice, hepatic Th1 responses developed quite slowly from d14 p.i. on following *L. donovani* inoculation and peaked between d21 and 28 p.i., when about 20% of the CD4 T cells expressed IFNγ. Similarly to *Irf5*^−/−^ mice (23), infected CMV-*Cre*^+^ mice generated defective Th1 responses and only about 10% of the CD4 T cells in the liver were IFNγ^+^ at d 28 p.i. (Figures 1C,D; gating strategy is shown in Supplemental Figure 1A). Interestingly, myeloid-specific *Irf5*^−/−^ mice had similar frequencies of IFNγ^+^ CD4 T cells to the *Cre*^−^ controls, suggesting that IRF-5 expression in myeloid cells is not required for Th1 development in *L. donovani* infected mice.

### *Irf5^*flox*/*flox*^*- *LysM-Cre^+^* Mice Develop Splenomegaly

**Figure 2:**
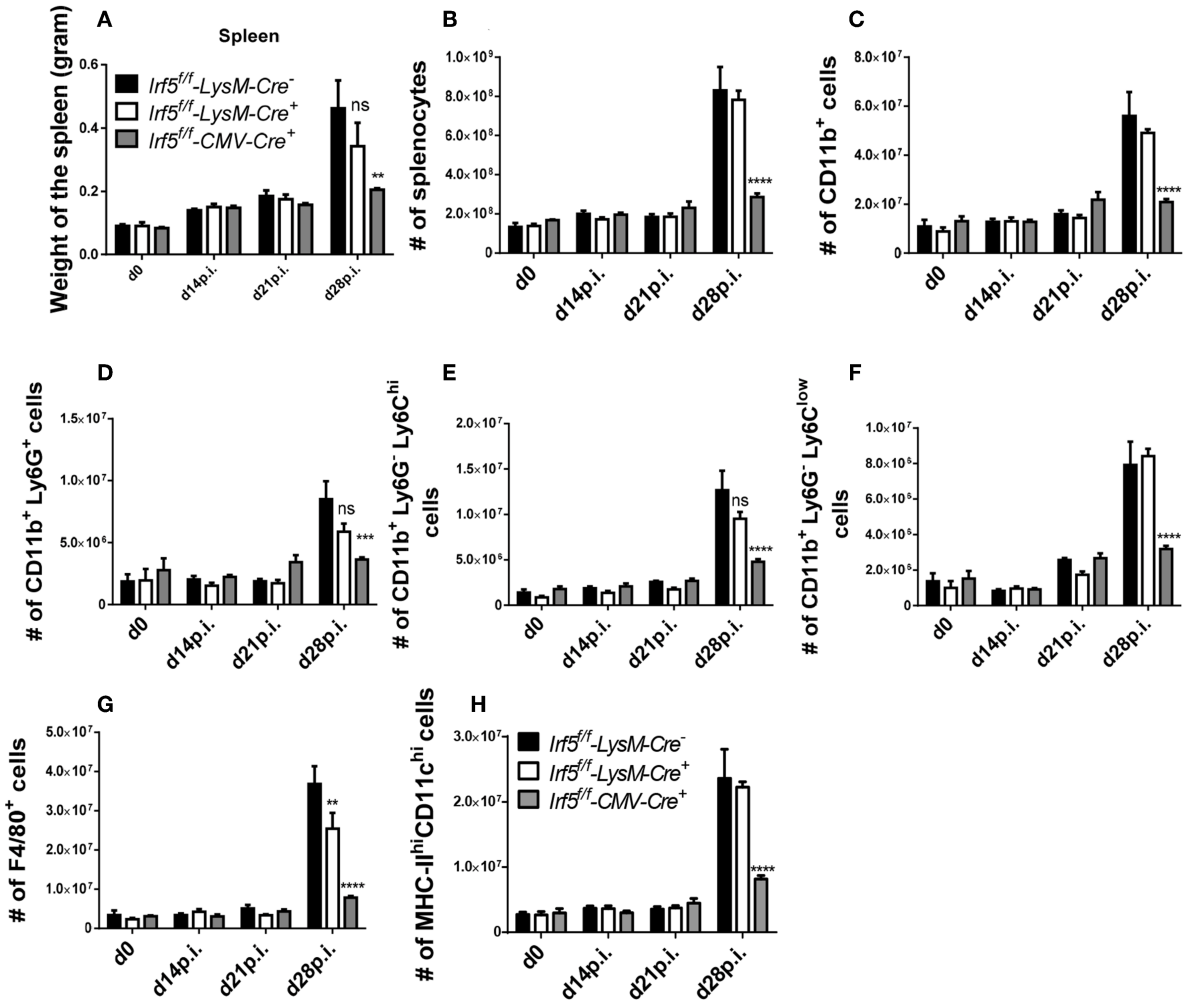
*Irf5*^*f*/*f*^ -*LysM-Cre*^+^ mice develop splenomegaly following *L. donovani* infection. *Irf5*^*f*/*f*^ **-***LysM-Cre*^−^, *Irf5*^*f*/*f*^ **-***LysM-Cre*^+^, and *Irf5*^*f*/*f*^ **-***CMV-Cre*^+^ mice were infected with *L. donovani* and sacrificed at d14, d21, and d28 p.i. Graphs show spleen weights **(A)**, number of splenocytes **(B)** and absolute number of splenic CD11b^+^ cells **(C)** in naïve and infected mice over the course of infection. Splenocytes were stained with different surface markers; neutrophils were excluded before analyzing monocytes and monocyte-derived cells. Graphs show absolute number of Ly6G^+^ neutrophils **(D)**, Ly6C^high^ monocytes **(E)**, Ly6C^low^ monocytes **(F)**, F4/80^+^ cells **(G)**, and MHC-II^high^ CD11c^high^ cells **(H)**. Data is shown as the mean ± SEM, *N* = 3–4, one of 5 independent experiments is shown, ns, not significant, **p < 0.01, ***p < 0.001, ****p < 0.0001.

Next, we wanted to determine whether IRF-5 expression in myeloid cells was at all required to induce splenomegaly that is typically present following *L. donovani* infection. As expected, the splenic weight (Figure 2A) and cellularity (Figure 2B) constantly increased over the course of *L. donovani* infection in *Cre*^−^ controls. In contrast, spleen of infected CMV-*Cre*^+^ mice had a significantly lower weight (Figure 2A) and cell count (Figure 2B), in agreement with our previously published results using *Irf5*^−/−^ (23). Because myeloid cells are increasingly recruited to the spleen during *L. donovani* infection (14, 15), we analyzed myeloid cell recruitment over the course of infection in the three groups of mice. As shown in Figure 2C, the number of CD11b^+^ cells present in the spleen of *Cre*^−^ mice progressively increased until d28 p.i.; comparable myeloid cell numbers were also observed in the spleen of cell-specific *Irf5*^−/−^ mice (Figure 2C). In contrast, splenic myeloid cell numbers in total IRF-5-deficient mice (CMV-*Cre*) did not substantially vary over the course of disease and were significantly lower than in the other two groups (Figure 2C). We obtained similar results when we analyzed the various myeloid cell subpopulations. In naïve mice and in *L. donovani*-infected mice until d14p.i., splenic myeloid cells can be clearly subdivided into neutrophils (CD11b^hi^Ly6G^+^), inflammatory monocytes (CD11b^hi^Ly6G^−^Ly6C^hi^), non-classical monocytes (CD11b^hi^Ly6G^−^Ly6C^lo/int^), macrophages (CD11b^+^F4/80^+^MHCII^lo^), and conventional dendritic cells (CD11c^hi^MHCII^int^/^hi^) (gating strategies for all myeloid cell populations are in Supplemental Figure 1B). However, during chronic *L. donovani* infection these populations are much less clearly defined. With exception of neutrophils, which remain CD11b^hi^Ly6G^+^, at chronic stages of disease monocytes and monocytes-derived cells, such as macrophages and dendritic cells, acquire very similar markers, namely CD11b, MHCII, CD11c, and F4/80 (14, 15), which makes it very difficult to unmistakably differentiate dendritic cells and macrophages from monocytes and from each other. As observed for total myeloid cells, the number of neutrophils (Figure 2D), inflammatory monocytes (Figure 2E), non-classical monocytes (Figure 2F), CD11b^+^F4/80^+^ cells (corresponding to macrophages at d0 and 14p.i. and monocytes, macrophages and DCs at d21 and 28) (Figure 2G), and CD11c^hi^MHCII^hi^ (DCs at d0 and 14p.i and DC and inflammatory monocytes at d21 and 28p.i) (Figure 2H) dramatically increased during chronic stages of infection in IRF-5-sufficient control mice. As expected, the numbers of splenic neutrophils, monocytes, macrophages and DCs did not substantially change over the course of infection in *CMV-Cre*^+^ mice and were significantly lower than those observed in *Cre*^−^ mice (Figures 2D–H). Surprisingly, no significant differences were observed in the numbers of splenic myeloid cell populations, with exception of F4/80^+^ cells that were slightly fewer in cell-specific *Irf5*^−/−^ mice at d28p.i. when compared with IRF-5 sufficient control mice (Figures 2D–H). No differences were observed among the three groups of mice in the percentage of the various myeloid cell populations (data not shown), suggesting that the defect in recruitment observed in IRF-5-deficient mice at d 28 p.i. was not confined to myeloid cells.

Taken together, our results imply that IRF-5 expression in myeloid cells is not required for promoting inflammatory cell infiltration, Th1 responses, and splenomegaly during chronic experimental VL.

### *LysM-Cre*^−^ Fails to Delete *Irf5* in Splenic Myeloid Cells During Chronic VL

**Figure 3:**
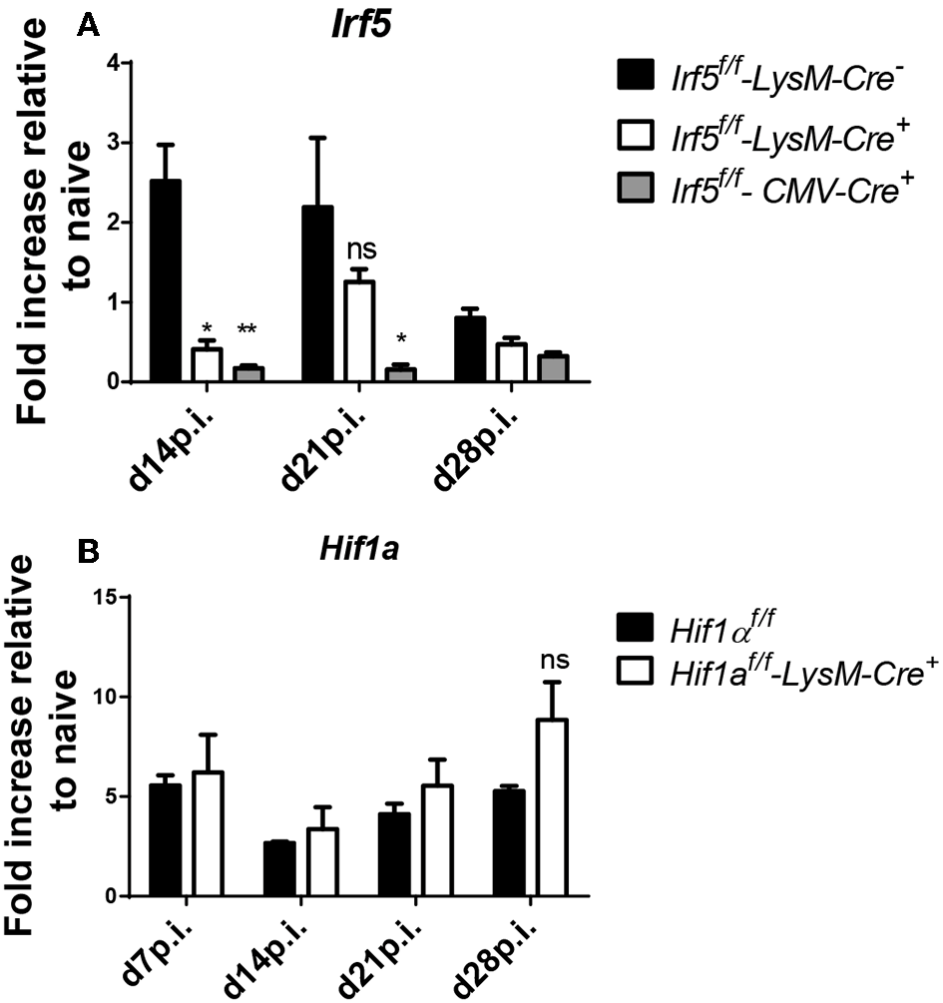
*LysM-Cre*^+^ failed to delete *Irf5* and *Hif1*α in splenic myeloid cells during *L. donovani* infection. **(A)** Real time PCR analysis of *Irf5* mRNA expression in splenic myeloid cells isolated from naive and infected *Irf5*^*f*/*f*^**-***LysM-Cre*^−^, *Irf5*^*f*/*f*^**-***LysM-Cre*^+^, and *Irf5*^*f*/*f*^**-***CMV-Cre*^+^ mice, expressed as fold increase compared to naïve mice. **(B)** Real time PCR analysis measuring *Hif1*α mRNA expression in myeloid cells isolated from the spleen of naive and infected *Hif1*α^*f*/*f*^ and *Hif1*α^*f*/*f*^-*LysM-Cre*^+^ mice and expressed as fold increase compared to naïve mice. Data represents mean ± SEM of 2 experiments, *N* = 8, ns, not significant; *p < 0.05, **p < 0.01.

Although *LysM-Cre* mice are the model of choice to delete specific genes in myeloid cells, we were puzzled by the fact that deletion of *Irf5* in myeloid cells did not have any effect at all on the immune response to *L. donovani* or on the course of infection. Hence, we suspected that the cre recombinase had low deletion efficiency in splenic cells expressing lysozyme 2 in our particular infection model. Hence, we analyzed *Irf5* expression by qPCR in purified splenic myeloid cells in *L. donovani* infected *Cre*^+^ and *Cre*^−^ mice. As a control, we also measured *Irf5* mRNA expression levels in CD11b^+^ cells from *L. donovani* infected *Irf5-CMV-Cre*^+^ mice, which, as expected, failed to upregulate *Irf5* (Figure 3A). Significant deletion in CD11b^+^ cells was only achieved at d14p.i. (Figure 3A); however, no difference in *Irf5* mRNA expression was noted at d21 between *Cre*^+^ and *Cre*^−^ mice (Figure 3A). To exclude the possibility that this lack of deletion was associated with our particular target gene, *Irf5*, or our *Irf5*^*flox*/*flox*^ mice, we tested cre recombinase efficiency in splenic myeloid cells in a different mouse model, using a different target gene, namely *Hif1a*. Like for *Irf5*, we analyzed *Hif1a* mRNA expression in purified splenic myeloid cells over the course of *L. donovani* infection in *Hif1a*^*flox*/*flox*^ – *LysM-Cre*^+^ and *Cre*^−^ mice. As shown in Figure 3B, no differences were observed in *Hif1a* expression in splenic CD11b^+^ cells from both groups of mice at any time point of infection, suggesting that the *LysM-Cre* model cannot be used to delete genes in splenic myeloid cells in the context of experimental VL.

### Myeloid Cell-Derived IRF-5 Promotes Splenomegaly but Is Not Required to Induce Th1 Responses During *L. donovani* Infection

**Figure 4:**
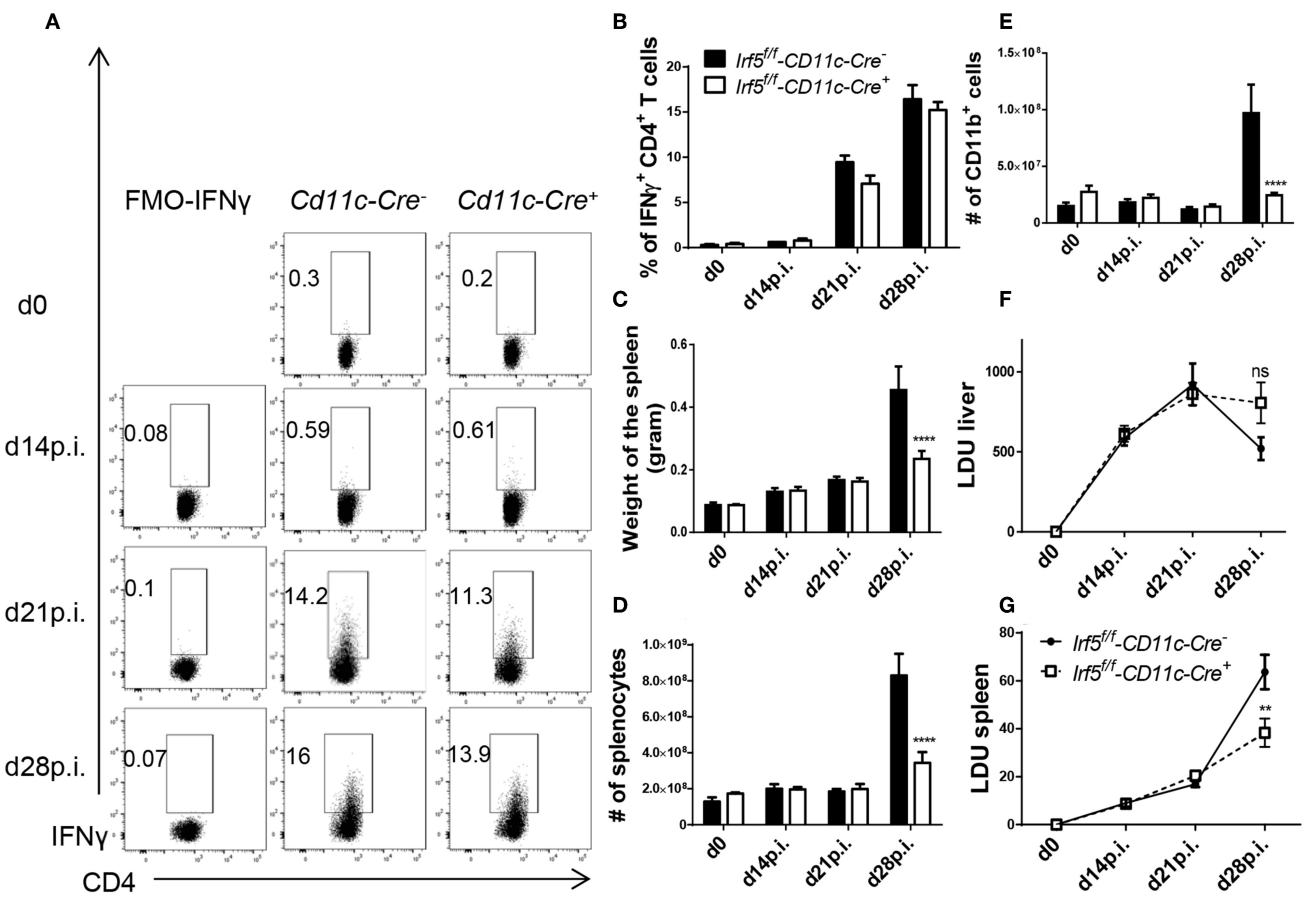
*Irf5*^*f*/*f*^ -*CD11c-Cre*^+^ mice fail to develop splenomegaly but show similar frequencies of Th1 cells than *Irf5*^*f*/*f*^ -*CD11c-Cre*^+^ mice following *L. donovani* infection. *Irf5*^*f*/*f*^ -*CD11c-Cre*^+^ and *Irf5*^*f*/*f*^ -*CD11c-Cre*^−^ mice were infected with *L. donovani* and sacrificed at various time points of infection. Graphs show representative scatter plots **(A)** and percentages **(B)** of IFN-γ^+^ CD4 T in the liver of naïve and infected mice. Graphs show spleen weights **(C)**, absolute number of splenocytes **(D)** and splenic CD11b^+^ cells **(E)** in naïve and infected mice. **(F,G)** Graphs show hepatic **(F)** and splenic **(G)** parasite burden, expressed as Leishman Donovan Units. Error bars indicate mean ± SEM, *N* = 4 mice, one of 2 independent experiments is shown, ns, not significant, **p < 0.01, ****p < 0.0001.

We next turned our attention to another model that we successfully used in the past to delete *Hif1a* in splenic myeloid cells during *L. donovani* infection (14), namely knock-in mice expressing the cre recombinase under the CD11c promoter. This model can be used for experimental VL infection, since most splenic myeloid cells, excluding neutrophils, express CD11c during chronic infection and the deletion efficiency is much higher than that achieved with *LysM-Cre* mice (14). Thus, we generated *Irf5*^*flox*/*flox*^ -*Cd11c-Cre*^+^ and *Cre*^−^ mice and infected them with *L. donovani*. First we investigated the development of IFNγ^+^ CD4 T cell responses in the liver, as those were mostly affected in total *Irf5*^−/−^ mice following *L. donovani* infection. Surprisingly and in disagreement with the literature, we found that *Irf5* deletion in CD11c^+^ cells did not affect the Th1 immune response; indeed, comparable frequencies of IFNγ^+^ CD4 T cells were observed at various time points after infection in the liver of both groups of mice (Figures 4A,B; gating strategy in Supplemental Figure 1A). Similarly, no differences were observed in the frequency of splenic IFNγ^+^ CD4 T cells between both groups of mice (Supplemental Figure 2).

However, *Irf5*^*flox*/*flox*^ -*Cd11c-Cre*^+^ mice had a much smaller spleen at d28p.i. in weight (Figure 4C) and cellularity (Figure 4D), compared with their *Cre*^−^ counterparts, suggesting that IRF-5 expression in CD11c^+^ cells is required to promote inflammation in the spleen following *L. donovani* infection. Moreover, myeloid cell numbers in *L. donovani*-infected *CD11c-Cre*^+^ mice were significantly reduced at d28 p.i. (Figure 4E). This lack of recruitment affected all myeloid cell populations (Supplemental Figures 3A–E). Similarly to *Irf5*^*flox*/*flox*^ -*CMV-Cre*^+^ mice, reduced recruitment of inflammatory cells affected all cell populations equally; percentages of B, T, and myeloid cells did not vary between *CD11c-Cre*^+^ and *Cre*^−^ mice (data not shown).

Because the recruitment of monocytes has been associated with disease exacerbation (15), we were curious to know whether a dramatic reduction in inflammatory monocyte infiltration to the spleen would affect the parasite burden. As shown in Figure 4F, no significant differences in LDU were observed in the livers of infected *Cre*^+^ and *Cre*^−^ mice. In contrast, the splenic parasite burden was significantly lower in the absence of *Irf5* expression in CD11c^+^ cells (Figure 4G).

In summary, our results show that IRF-5-deficiency in CD11c^+^ cells severely affects inflammatory cell infiltration to the spleen, but does not hinder the development of Th1 responses in *L. donovani*-infected mice.

## Discussion

Splenomegaly and chronic inflammation are hallmark of visceral leishmaniasis and are associated with parasite persistence. IRF-5 is largely responsible for promoting inflammatory cell infiltration into the spleen, which ultimately results in splenomegaly. In the present study, we show that *Irf5* expression in CD11c^+^ cells is involved in the induction of inflammation but is not necessary for development and/or maintenance of protective Th1 responses.

The *LysM-Cre* mouse model has successfully been used to study the role of specific genes in myeloid cells in the bone marrow (28), the lungs (29), the liver (30), the gut and the skin (31, 32). However, LysM-cre does not seem to be effective in deleting genes in splenic myeloid cells during VL. Our results are in agreement with Abram et al. that report deletion efficiency for LysM-cre of less than 40% in splenic macrophages and blood monocytes (33). Furthermore, analysis of mice with an insertion of EGFP into the *LysM* gene revealed that LysM is mostly highly expressed in granulocytes, especially neutrophils, followed by macrophages and only occasionally by monocytes (34). Thus, it is not surprising that this mouse model cannot be used to study gene functions in splenic myeloid cells following *L. donovani* infection, with exception of neutrophils. One should also take into account that deletion efficiency in blood monocytes is also very low and that this may change the interpretation of studies in disease models with extensive monocyte infiltrations, like cutaneous leishmaniasis for example. In an earlier publication, we have found that the *CD11c-Cre* mouse model is a better choice for deleting genes in splenic myeloid cells in the context of VL (14), because of the massive infiltration of inflammatory monocyte, which express high levels of CD11c during the chronic stage of disease. The current results confirm our previous data. Indeed, we observed a clear phenotype in CD11c-specific *Irf5*^−/−^ mice after *L. donovani* infection, suggesting that *Irf5* expression in neutrophils does not play a major role in the immunopathogenesis of VL.

Interestingly, *L. donovani*-infected CD11c-specific *Irf5*^−/−^ mice display a massive impairment of myeloid cell recruitment to the spleen compared to IRF-5-sufficient mice. Splenomegaly results from the induction of emergency hematopoiesis, mainly during the chronic stages of *L. donovani* infection (14, 15). The fact that neutrophils, which are IRF-5 sufficient in our model, were also not recruited to the spleen suggest that IRF-5 may be required to drive inflammation/emergency hematopoiesis and/or for cell migration. In an experimental model of SLE, IRF-5 was reported to regulate the expression of CXCR4 and CCR2, two important molecules involved in monocyte migration (35). Moreover, IRF-5 is essential for maintaining pro-inflammatory CD11c^+^ macrophages/monocytes within lesions in an experimental atherosclerosis model (36). It is also possible that *Irf5* expression in B cells may be involved in initiating inflammation. In fact, IRF-5 regulates activation, proliferation, differentiation and antibody production in human naïve B cells (37); moreover, *Irf5*^−/−^ mice showed a reduction in hypergammaglobulinemia (38), which is required for sustaining inflammation during VL (39). Further investigations are needed to determine the functional role of this transcription factor in driving inflammation during VL. Taken together, our data demonstrate that a reduction in the recruitment of myeloid cells to the spleen during chronic infection results in heightened host resistance against *L. donovani*. These results are in agreement with the literature that reports a detrimental role for myeloid cells, particularly inflammatory monocytes, during VL (14, 16, 40).

We were surprised to observe that Th1 responses were not altered in myeloid cell-specific *Irf5*^−/−^ mice. Although T cell recruitment to the spleen was also impaired in CD11c-specific *Irf5*^−/−^ mice during VL, the frequency of IFNγ^+^ CD4 T cells was comparable to *Cre*^+^ mice, suggesting that *Irf5* expression in CD11c^+^ cells is not required for sustaining Th1 responses. This disagreement with the literature (27) could be explained by the fact that CD4 T cell responses are primed very late during infection and peak at d21–28 p.i., when IRF-5 is not strongly expressed by myeloid cells. It is thus important to integrate knowledge on myeloid cell functions with the kinetic of CD4 T cell priming and development in order to identify crucial molecules involved in these processes for a particular infection. IRF-5 expression in CD11c^+^ cells is not essential for Th1 priming during VL.

Because Th1 responses are thought to be essential for controlling parasite growth in leishmaniasis, it still remains unclear why the splenic parasite burden was lower in *Cd11c-Cre*^+^ mice compared to *Cre*^−^ controls. One possible explanation is that monocytes, which act as safe houses during chronic VL (14–16), are recruited in lower numbers to the spleen. A second possibility that is not mutually exclusive with the first one is that CD8 T cells undergo stronger expansion in these mice. We have previously reported that IRF-5-mediated inflammation was required to induce HIF-1α expression in dendritic cells and that this had an negative impact on their function and on CD8 T cell expansion during the first 10 days of infection (6). It is thus possible that CD8 T cells underwent stronger expansion in infected *Cd11c-Cre*^+^ mice and that this contributed to enhanced parasite growth control.

In conclusion, we demonstrated that *Irf5* expression in CD11c^+^ cells is required for the development of splenomegaly but does not affect the development or the maintenance of protective Th1 responses during experimental VL. Moreover, the *Lysm-Cre* mouse model should not be used to delete genes in splenocytes or inflammatory monocytes infiltrating the spleen.

## Data Availability Statement

All datasets generated for this study are included in the article/Supplementary Material.

## Ethics Statement

The animal study was reviewed and approved by Comité Institutionnel de Protection des Animaux of the INRS-Institut Armand Frappier.

## Author Contributions

LM designed and performed experiments, analyzed data, wrote the manuscript. MS, SS-B, and AF performed experiments and analyzed data. SS directed the study, interpreted data, and wrote the manuscript.

### Conflict of Interest

The authors declare that the research was conducted in the absence of any commercial or financial relationships that could be construed as a potential conflict of interest.
