# Supplementary material for: IRF-5 Expression in Myeloid Cells Is Required for Splenomegaly in L. donovani Infected Mice
Source: Front Immunol. 2020 Jan 21;10:3071. doi: 10.3389/fimmu.2019.03071 (PMC6985270; doi:10.3389/fimmu.2019.03071)
Supplement: Supplementary file 1 [file Presentation_1.pdf]

Supplemental Figure 1

A

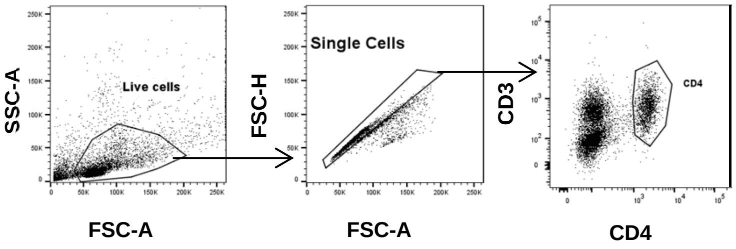


Gating strategy for figures 1D and 4A

B

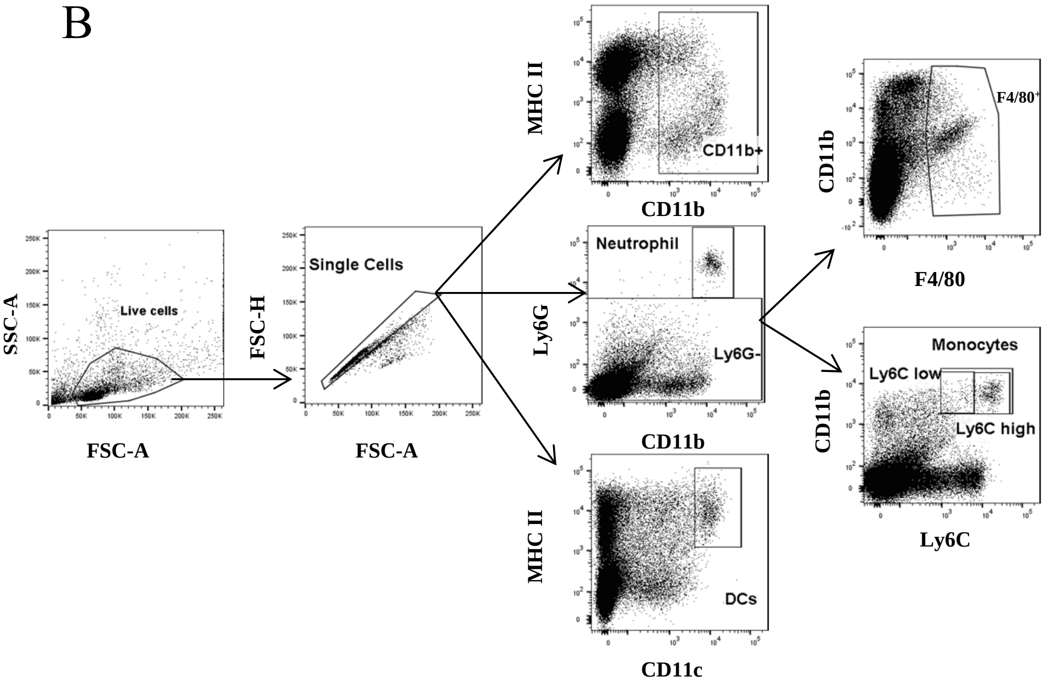


Gating strategy for figure 2

Supplemental Figure 2

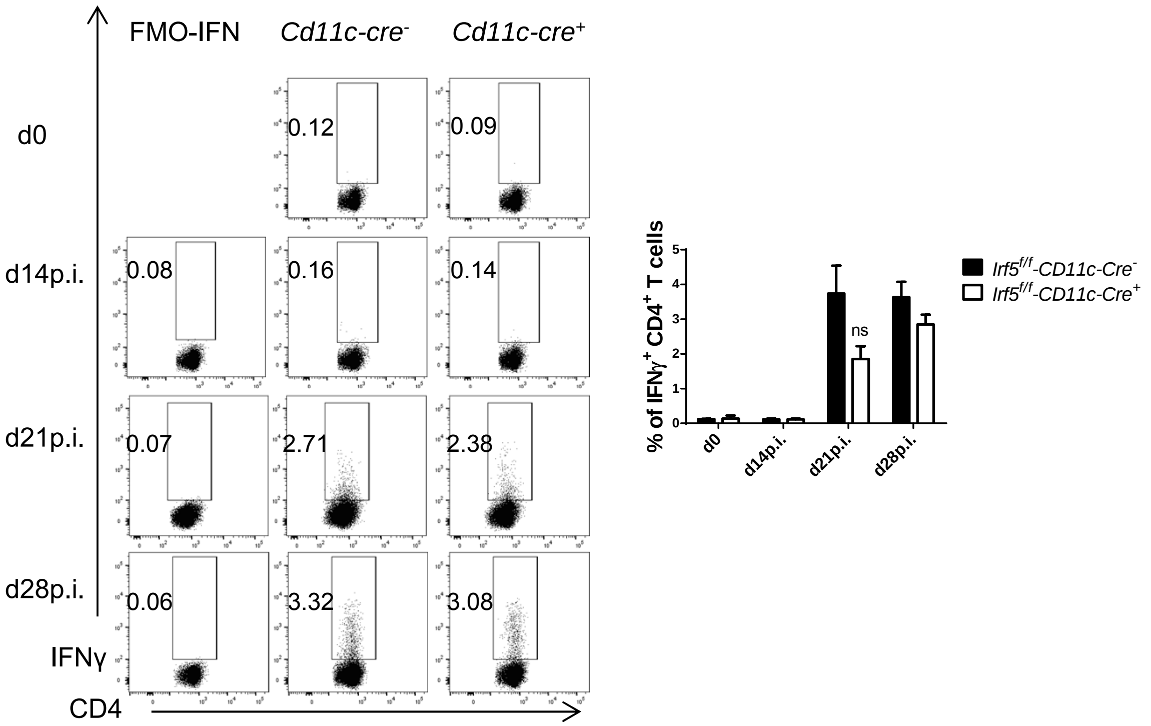


**Figure 2. *L. donovani* infected *Irf5-Cd11c-Cre*+ and *Cre*- mice develop similar Th1 responses in the spleen.** *Irf5-Cd11c-Cre*+ and *Cre*- mice were infected with *L. donovani* and sacrificed at different time points. Splenocytes were restimulated with bone marrow-derived DCs pulsed with fixes *L. donovani* amastigotes O/N for six hours in the presence of Brefaldin A. Cells were then stained for CD3 and CD4, and with anti-IFNγ after fixation and permeabilization. Graphs show the percentage of IFNγ+ CD4 T cells in both groups of mice. ns denotes not significant.

# Supplemental Figure 3

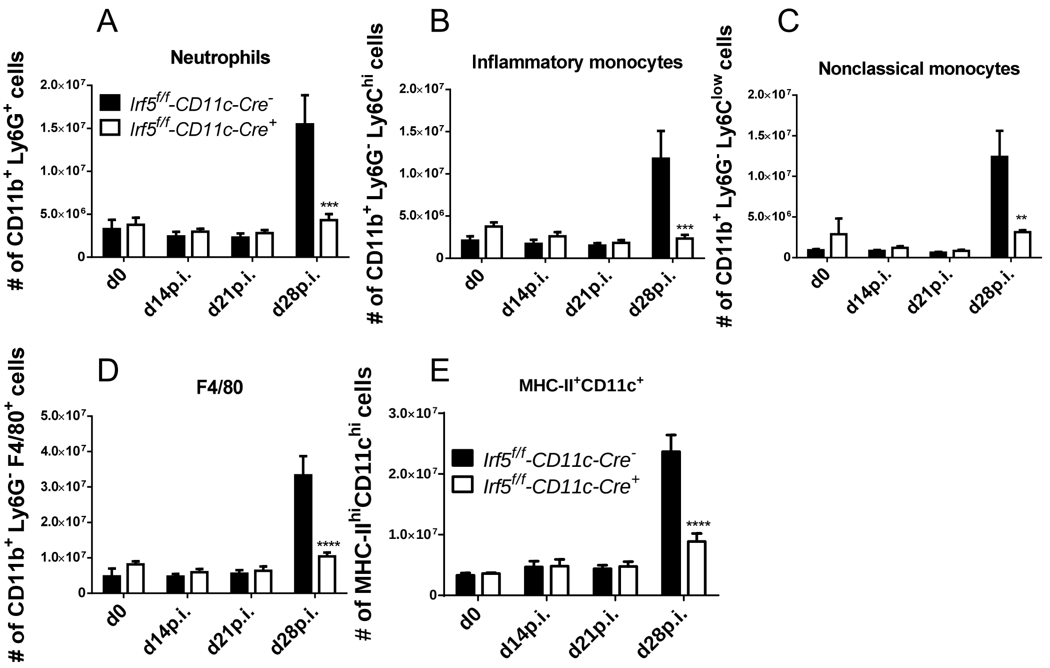


**Figure 3. The migratory defect observed in *Irf5$^{f/f}$-Cd11c-Cre$^{+}$* mice during VL affects all splenic myeloid cell populations.** *Irf5$^{f/f}$-Cd11c-Cre$^{+}$* and *Irf5$^{f/f}$-Cd11c-Cre$^{-}$* mice were infected with *L. donovani* and sacrificed at different time points p.i. Splenocytes were stained with different surface markers; neutrophils were excluded before analyzing monocytes and monocyte-derived cells. Graphs show absolute numbers of splenic Ly6G$^{+}$ neutrophils (A), Ly6C$^{hi}$ inflammatory monocytes (B), Ly6C$^{lo}$ non-classical monocytes (C), F4/80$^{+}$ cells (D), and MHC-II$^{hi}$ CD11c$^{hi}$ cells (F). Data is shown as the mean ± SEM, n=3-4, ns denotes not significant, ** denotes $p<0.01$, *** denotes $p<0.001$, **** denotes $p< 0.0001$
